# Anesthesia experience in an adult Silver-Russell syndrome: a case report

**DOI:** 10.1186/s40981-024-00704-5

**Published:** 2024-04-03

**Authors:** Akinobu Hibino, Ayaka Hibino, Yoshinori Kamiya

**Affiliations:** 1 Department of Anesthesiology, Shibata Hospital Niigata Prefectural Hospital, 1-2-8 Honcho, Shibata City, Niigata 957-8588 Japan; 2 Department of Anesthesiology, Niigata University Medical and Dental Hospital, 1-754 Asahimachi-Dori, Chuo-Ku, Niigata City, Niigata 951-8510 Japan; 3 Department of Anesthesiology and Pain Medicine, Gifu University Graduate School of Medicine, 1-1 Yanagido, Gifu City, Gifu 501-1194 Japan

**Keywords:** Russell-Silver syndrome, Epidural anesthesia, Percutaneous endoscopic gastrostomy, Carbon dioxide narcosis, Difficult airway management

## Abstract

**Background:**

There are no reports of anesthesia use in adult patients with Silver-Russell syndrome (SRS). Here, we report our experience with anesthesia in an adult patient with SRS complicated by chronic respiratory failure.

**Case presentation:**

A 33-year-old woman was clinically diagnosed with SRS. She had severe chronic respiratory failure, complicated by superior mesenteric artery syndrome. Percutaneous gastrostomy was scheduled for nutritional management under epidural anesthesia; however, soon after esophagogastroduodenoscopy was started, she lost consciousness and spontaneous respiration. The patient was urgently intubated and converted to general anesthesia. The end-tidal carbon dioxide tension was > 90 mmHg at intubation.

**Conclusions:**

Adult SRS patients with chronic respiratory failure have a risk of CO_2_ narcosis. SRS also requires preparation for difficult airway management during the perioperative period.

## Background

Silver-Russell syndrome (SRS) is characterized by intrauterine growth retardation, short postnatal stature and weight, skeletal asymmetry, and an inverted triangular face with a large head [1]. Gastrointestinal disorders are common features of this syndrome [2]. Anesthesia management of SRS is reported only in pediatric cases. We report the anesthetic management of an adult patient with SRS complicated by severe chronic respiratory failure and superior mesenteric artery (SMA) syndrome [3]. The patient was scheduled for percutaneous gastrostomy (PEG) under epidural anesthesia but was urgently intubated and converted to general anesthesia due to intraoperative CO_2_ narcosis. This is a case report of an anesthetic experience in an adult patient with SRS.

## Case presentation

**Fig. 1:**
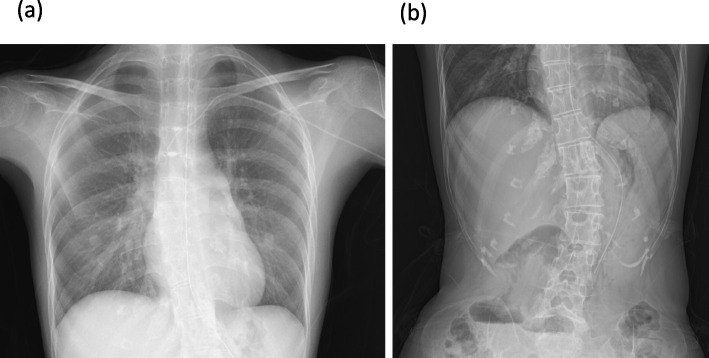
Preoperative radiographs of **a** chest and **b** abdomen. There was scoliosis of the thoracolumbar spine and whole-body muscle atrophy, but no severe thoracic deformity

A 33-year-old woman (weight, 16.8 kg; height, 131 cm; BMI 9.8 kg/cm^2^) with Silver-Russell syndrome (SRS) experienced hypercapnic respiratory failure [3]. Computed tomography scans showed muscle atrophy, including respiratory muscles, and SMA syndrome. Hospitalized for chronic respiratory failure, she started noninvasive ventilation after losing consciousness from CO_2_ narcosis. Respiratory muscle weakness, worsened by SRS and malnutrition from SMA syndrome, caused further decline. Despite receiving nasal nutrition and central venous catheter support, she developed aspiration pneumonia, aggravating her hypercapnia. Preoperative evaluation revealed severely restricted pulmonary function with her vital capacity of 0.40 L (17% of the predicted value) and thoracolumbar scoliosis without major thoracic deformity Fig. 1. Arterial blood gases (ABG) in ambient air indicated significant respiratory acidosis; pH of 7.28, pCO_2_ of 75.7 mmHg, pO_2_ of 93.4 mmHg, and HCO_3_^−^ of 34.8 mEq/L. Esophagogastroduodenoscopy (EGD) for PEG planning was uneventful. Due to the high CO_2_ narcosis risk from intraoperative sedation and expected ventilator weaning challenges, PEG was scheduled under epidural anesthesia.

The patient underwent epidural anesthesia. Positioned in left lateral decubitus, the epidural puncture was performed at Th8-9, reaching a depth of 2 cm. An epidural catheter was inserted 3 cm into the epidural space. Initial anesthesia involved injecting 3 mL of 1% mepivacaine, with the anesthetic area confirmed by a cold test at Th8-9 after approximately 3 min. Subsequently, 3 mL of 2% mepivacaine was administered for extended anesthesia, and the anesthetic area was verified by a cold test at Th5-12.

After ensuring sufficient anesthesia, a gastroenterologist performed an EGD with CO_2_ insufflation. Within minutes of endoscope insertion, the patient lost consciousness and spontaneous breathing. The endoscope was promptly removed, and mask ventilation was initiated. As the patient did not recover, a decision was made to perform tracheal intubation for ventilatory management. Anticipating intubate difficulties, a difficult airway management (DAM) cart was prepared. Adequate oxygenation was maintained with mask ventilation, FiO_2_ 1.0, and a tidal value of around 150 mL. General anesthesia was induced using a continuous infusion of the target-controlled infusion of 1% propofol 4 mcg/mL and 0.3 mcg/kg/min remifentanil, with muscle relaxation achieved by administering 20 mg of rocuronium. Laryngeal visualization was straightforward with the percentage of the glottic opening score was 100% using a McGRATH® blade #3, and successful tracheal intubation was accomplished with a 7.0 mm tracheal tube. End-tidal carbon dioxide levels exceeded 90 mmHg at intubation. Post-intubation, oxygen, and air were administered, and general anesthesia was maintained with reduced doses of propofol and remifentanil. Continuous monitoring was done with an arterial line, blood glucose level remained at 100 mg/dL and there was no hypoglycemia. In consultation with a gastroenterologist, it was decided to proceed with surgery under general anesthesia. Propofol and remifentanil were discontinued as the surgery was completed, and 50 mg of sugammadex was administered. Spontaneous respiration resumed upon awakening. The patient was extubated after confirming stable breathing and observed in the operating room for approximately 30 min, the result of ABG with O_2_ 3 L/min mask administration showed as below; pH of 7.34, pCO_2_ of 70.3 mmHg, pO_2_ of 96.3 mmHg, and HCO_3_^−^ of 37.7 mEq/L. Subsequently, she was transferred to the intensive care unit for respiratory management. She was discharged 2 days later as oxygenation could be maintained with nasal cannula administration of 2 L/min of oxygen.

## Discussion

Perioperative precautions for SRS include DAM. Gastrointestinal disorders and GERD are often associated with SRS [2]; tube feeding may be performed.

There have been numerous reports of difficult intubation in pediatric patients with SRS. Imaizumi et al. reported a case of pediatric SRS in which the patient was difficult to intubate due to mandibular maldevelopment and an elevated palate [4]. Anesthetic management of SRS in adults has not been reported, although it is necessary to anticipate and prepare for difficult intubation, as in pediatric patients.

In this case, the patient with SMA syndrome had a feeding disorder and was reliant on nasotracheal tube feeding, mask ventilation and tracheal intubation were not difficult in this patient. She had respiratory muscle atrophy due to developmental delays from SRS, weakness from malnutrition and SMA syndrome, resulting in chronic respiratory failure and hypercapnia. PEG, performed under EGD with local anesthetics and sedatives, posed a high risk of CO_2_ narcosis due to respiratory depression from sedation. Consequently, perioperative management aimed to preserve spontaneous respiration and minimize airway intervention. The patient's mild scoliosis allowed for epidural anesthesia, which provided an adequate anesthetic range for the surgery, thus avoiding general anesthesia.

The patient experienced CO_2_ narcosis immediately after EGD insertion, despite the absence of such episodes in prior EGD procedures. In Japan, CO_2_ insufflation is commonly employed during EGD and is considered as safe as air insufflation. Nonaka et al. reported that CO_2_ insufflation during EGD does not lead to a significant rise in transcutaneous CO_2_ monitoring [5]. They attribute this to CO_2_ quickly absorbing from the intestines, along with slight abdominal distension and diaphragmatic elevation, which reduce restrictions on ventilation volume and rate.

Nevertheless, in patients with severe chronic respiratory failure, the CO_2_ introduced during EGD was quickly absorbed from the gastrointestinal tract, dissolved in the blood, and exceeded acceptable levels for blood CO_2_. Additionally, managing the patient under epidural anesthesia created conditions where CO_2_ narcosis could easily occur. While hypotension is commonly emphasized in epidural anesthesia, it is also recognized that respiratory muscle weakness can result when the anesthesia extends to the upper thoracic region, potentially suppressing respiratory function [6].

Typically, respiratory issues from epidural anesthesia are not clinically concerning. In this patient with chronic respiratory failure from respiratory muscle atrophy, even mild motor inhibition from epidural anesthesia decreased ventilatory capacity, impairing CO_2_ elimination. A decrease in minute ventilation led to increased blood CO_2_, resulting in CO_2_ narcosis in this case.

Contrastingly, loss of consciousness and respiratory arrest symptoms indicated hypoglycemia and local anesthesia toxicity. However, ABG revealed no hypoglycemia and initial ECG monitoring showed no strong signs of local anesthesia toxicity. Alternatives like EGD with air insufflation or small laparotomy may be better for epidural anesthesia. Further consideration should have been given to the possibility of administering a peripheral nerve block in the abdomen. In the preparation process, alongside the DAM cart, it would have been necessary to procure a transcutaneous CO_2_ monitor and a muscle relaxation monitor. Contrary to our expectations, tracheal intubation was easily performed in this patient. The risk of DAM is high in pediatric patients with SRS; however, this has not yet been reported in adults. SRS has abnormal imprinted gene expression due to hypomethylation of the 11p15.5 region on chromosome 11 and chromosome 7 maternal dysomy, which induces abnormal skeletal development, resulting in DAM. Whole genome analysis of this case did not point to any genetic mutation characteristic of SRS, only mutations in the ALPL gene. Thus, it is unclear whether the results in other cases were similar to ours.

Most reports on anesthetic management of SRS are in pediatric cases. As there is limited literature on the long-term course of SRS, this is a rare case of adult SRS [3]. In this unique adult SRS case, we combined epidural and general anesthesia. Adults with SRS and chronic respiratory failure are prone to CO_2_ narcosis, even during minimally invasive EGD surgery.

## Conclusion

This is a case report on anesthetic encounters in adult SRS. Adults with SRS and chronic respiratory failure due to respiratory muscle atrophy face an increased risk of CO_2_ narcosis. Emergency DAM preparation is crucial for anesthesia administration.

## Data Availability

Not applicable.

## Abbreviations

SRS: Silver-Russell syndrome
SMA: Superior mesenteric artery
PEG: Percutaneous gastrostomy
ABG: Arterial blood gases
EGD: Esophagogastroduodenoscopy
DAM: Difficult airway management
